# Yin-Yang Mechanisms Regulating Lipid Peroxidation of Docosahexaenoic Acid and Arachidonic Acid in the Central Nervous System

**DOI:** 10.3389/fneur.2019.00642

**Published:** 2019-06-18

**Authors:** Bo Yang, Kevin L. Fritsche, David Q. Beversdorf, Zezong Gu, James C. Lee, William R. Folk, C. Michael Greenlief, Grace Y. Sun

**Affiliations:** ^1^Department of Chemistry, University of Missouri, Columbia, MO, United States; ^2^Department of Nutrition and Exercise Physiology, University of Missouri, Columbia, MO, United States; ^3^Departments of Radiology, Neurology and Psychological Sciences, and the Thompson Center, Columbia, MO, United States; ^4^Department of Pathology and Anatomical Sciences, University of Missouri, Columbia, MO, United States; ^5^Department of Bioengineering, University of Illinois at Chicago, Chicago, IL, United States; ^6^Biochemistry Department, University of Missouri, Columbia, MO, United States

**Keywords:** arachidonic acid, docosahexaenoic acid, cPLA_2_, iPLA_2_, lipid peroxidation, 4-hydroxyhexenal, 4-hydroxynonenal, neurodegeneration

## Abstract

Phospholipids in the central nervous system (CNS) are rich in polyunsaturated fatty acids (PUFAs), particularly arachidonic acid (ARA) and docosahexaenoic acid (DHA). Besides providing physical properties to cell membranes, these PUFAs are metabolically active and undergo turnover through the “deacylation-reacylation (Land's) cycle”. Recent studies suggest a Yin-Yang mechanism for metabolism of ARA and DHA, largely due to different phospholipases A_2_ (PLA_2_s) mediating their release. ARA and DHA are substrates of cyclooxygenases and lipoxygenases resulting in an array of lipid mediators, which are pro-inflammatory and pro-resolving. The PUFAs are susceptible to peroxidation by oxygen free radicals, resulting in the production of 4-hydroxynonenal (4-HNE) from ARA and 4-hydroxyhexenal (4-HHE) from DHA. These alkenal electrophiles are reactive and capable of forming adducts with proteins, phospholipids and nucleic acids. The perceived cytotoxic and hormetic effects of these hydroxyl-alkenals have impacted cell signaling pathways, glucose metabolism and mitochondrial functions in chronic and inflammatory diseases. Due to the high levels of DHA and ARA in brain phospholipids, this review is aimed at providing information on the Yin-Yang mechanisms for regulating these PUFAs and their lipid peroxidation products in the CNS, and implications of their roles in neurological disorders.

## Phospholipids in the Central Nervous System Are Enriched in ARA and DHA

**Figure 1:**
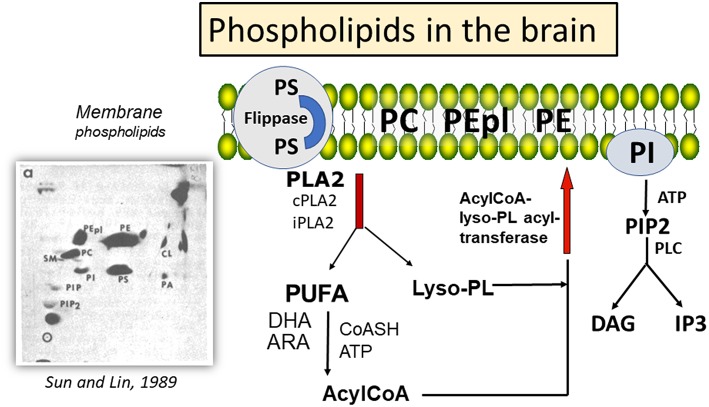
Mechanism for deacylation-reacylation of polyunsaturated fatty acids in phospholipids, and relative amount of phospholipids in the brain. ^a^High performance thin layer chromatography (HPTLC) separation of phospholipids in mouse cortex and detection by charring with cupric acetate; PE, phosphatidylethanolamine; PEpl, PE plasmalogen; PC, phosphatidylcholine; PS, phosphatidylserine; PI, phosphatidylinositol; PA, phosphatidic acid; Cl, cardiolipin; PIP, phosphatidylinositol-phosphate; PIP2, phosphatidylinositol 4,5-bisphosphate. HPTLC chromatograph was reprinted from Sun and Lin (5), with permission from Elsevier.

The mammalian brain is rich in lipids, with phospholipids playing important roles in biological functions. Changes in composition of membrane phospholipids can lead to alteration of cell functions, including signal transduction, cell-cell recognition, DNA replication, and protein trafficking (1), and with implications in neurological diseases, such as stroke, traumatic brain injury (TBI), depression (2), as well as Alzheimer's and Parkinson's diseases (AD and PD) (3, 4). Major phospholipids in brain include phosphatidylcholine (PC), phosphatidylethanolamine (PE), phosphatidylethanolamine plasmalogen (PEpl), together with smaller amounts of phosphatidylserine (PS), phosphatidylinositol (PI), and cardiolipin (CL) (5) (Figure 1). While these phospholipids are present in different types of cell membranes, CL is specifically present in the inner layer of mitochondrial membrane (6). Phospholipids have fatty acids (FAs) that are largely saturated or monoenes in the *sn-1* position of the glycerol moiety, whereas the *sn-2* position contains mainly polyunsaturated fatty acids (PUFAs). A characteristic feature for PE in brain is the large proportion of PEpl with alkenyl group in the *sn-1* position. These PEpl are abundant in the myelin sheaths (7). The PUFAs in PE are enriched in docosahexaenoic acid (22:6 n-3, DHA), whereas the PUFAs in PC have both DHA and arachidonic acid (20:4 n-3, ARA). PS is an anionic phospholipid with high levels of palmitic acid (16:0) and DHA, and translocation of this phospholipid from the inner to outer membrane surface through the flippases and scramblases can serve as an initiator for apoptotic processes through binding with annexin V (8, 9). PI is comprised of high levels of stearic acid (18:0) and ARA, and the inositol head group can be phosphorylated to form PIP and PIP2. Hydrolysis of PIP2 by phospholipase C results in the production of diacylglycerols and inositol phosphates (5), which are second messengers for activation of protein kinase C (PKC) and for mobilization of calcium from intracellular stores, respectively (10). An obvious difference between the PUFAs in the central nervous system (CNS) and the peripheral system is the low levels of linoleic acid (18:2 n-6) in CNS (11).

In the mammalian brain membranes, the PUFAs in the phospholipids (mainly PC and PE) are metabolically active and undergo turnover through the “deacylation-reacylation cycle”, also known as the “Land's cycle” (12, 13) (Figure 1). This cycle enables PUFAs to be released from membrane phospholipids through phospholipases A_2_ (PLA_2_s) and subsequently return to the membrane phospholipids through the lysophospholipid acyltransferases. In the CNS, different PLA_2_s are responsible for the release of DHA and ARA from phospholipids, thus suggesting a Yin-Yang mechanism for their metabolic functions (14). Besides production of eicosanoids and docosanoids, which are lipid mediators, these PUFAs are also substrates of oxygen free radicals, resulting in alkenal products that are metabolically active. In this review, attention is focused on factors regulating metabolism of ARA and DHA through different PLA_2_s, and the role of their peroxidation products in health and disease.

### ARA Release by cPLA_2_

As reviewed by Sun et al., release of ARA from phospholipids is catalyzed mainly by the Group IV calcium-dependent cytosolic PLA_2_α (cPLA_2_), a ubiquitous enzyme present in all cells in the CNS (15). Besides the requirement for calcium which binds to the C2 domain, a characteristic property of the cPLA_2_ is its susceptible to phosphorylation and activation by protein kinases, including the mitogen activated protein kinases (MAPKs) and PKC (16). A study with primary neurons demonstrated ability for NMDA (an excitatory glutamate receptor agonist) to stimulate phosphorylation of cPLA_2_ through activation of ERK1/2 (17). Studies with microglial cells also indicated the ability of lipopolysaccharides (LPS) to stimulate p-cPLA_2_ through p-ERK1/2 (18, 19).

Activation of cPLA_2_ and release of ARA have been implicated in a number of neurologic disorders and brain injury. Subjects with traumatic brain injury (TBI) showed a significant increase in PUFAs, including ARA, in the cerebrospinal fluid as compared with non-TBI controls (20, 21). In a study with acute stroke subjects, the decrease in DHA/ARA ratios in serum was attributed to the increase in cPLA_2_ activity and release of ARA (22, 23). Indeed, increases in p-cPLA_2_ were observed in animal models of cerebral ischemia (15). Furthermore, the transient increase in p-cPLA_2_ suggests tight regulation of this enzyme under pathological conditions (24).

A number of studies with animal models have demonstrated the involvement of cPLA_2_ in the pathophysiology of AD (25). Evidence included increases in ARA as well as its metabolites in the brain tissue of AD mutant mice (26). Studies with cultured neurons also demonstrated ability for the cytotoxic amyloid beta (Aβ) to stimulate phosphorylation of ERK1/2 and p-cPLA_2_ (17). Since action of cPLA_2_ not only releases PUFAs but also lysophospholipids, the increase in lysophosphatidylcholine (LPC) in AD mouse models was marked with a progressive decline in behavior (13). The changes in FAs as well as LPC are in agreement with perturbation of the “Land's cycle.” Using mass spectrometry (MS)-based shotgun lipidomics, analysis indicated increases in MAPK, PLA_2_, unesterified PUFAs, as well as LPC in the brains of AD transgenic mouse model (14). Using antisense nucleotide against cPLA_2_, studies by Levy's group demonstrated ability for aggregated Aβ to up-regulate cPLA_2_ in neurons and microglia (27, 28). Taken together, these studies demonstrate an important role for cPLA_2_ and the release of ARA in response to brain injury as well as in different neurologic disorders.

### The Release of DHA by iPLA_2_

The group VI calcium-independent PLA_2_s (iPLA_2_) are comprised of a number of isoforms and can release PUFAs (including ARA) from phospholipids. However, several studies suggested preference for the release of DHA through iPLA_2_β (29–32). As reviewed by Farooqui and Horrocks, iPLA_2_β is abundant in the brain, and is able to influence a number of CNS functions (33, 34). iPLA_2_-knockout mice showed a decrease in DHA metabolism, further demonstrating the association between DHA and iPLA_2_ (35). Recently, there is suggestion for a genetic link for this iPLA_2_ to PD (36). Mutation of iPLA_2_ was also linked to an infantile autosomal recessive gene disorder with brain iron accumulation, a neurologic disorder showing severe psychomotor and cognitive dysfunction (37, 38). The study by Shinzawa et al. showed neurologic impairments as well as accumulation of spheroids containing tubulovesicular membranes in a mouse model depleting this group of iPLA_2_ (39).

A study by Gattaz et al. indicated a significant decrease in activity of iPLA_2_ in platelets from AD patients and subjects with mild cognitive impairment (MCI) compared to normal controls (40). In fact, subjects with MCI also showed alterations of other types of PLA_2_, including cPLA_2_ and sPLA_2_. Chew et al. provided evidence for iPLA_2_ to play a role on synaptic plasticity and nociception, and these neuronal abnormalities could be reversed by suppressing iPLA_2_ through antisense oligonucleotide (41). Studies using hippocampal slices indicated involvement of iPLA_2_ in induction of long-term potentiation (42), and in agreement with the release of DHA through iPLA_2_, impairments in long-term potentiation could be reversed upon treatment with DHA (43, 44). Taken together, these studies provide support for the role of iPLA_2_ and DHA in modulating brain function.

## Lipid Mediators Derived From ARA and DHA

**Figure 2:**
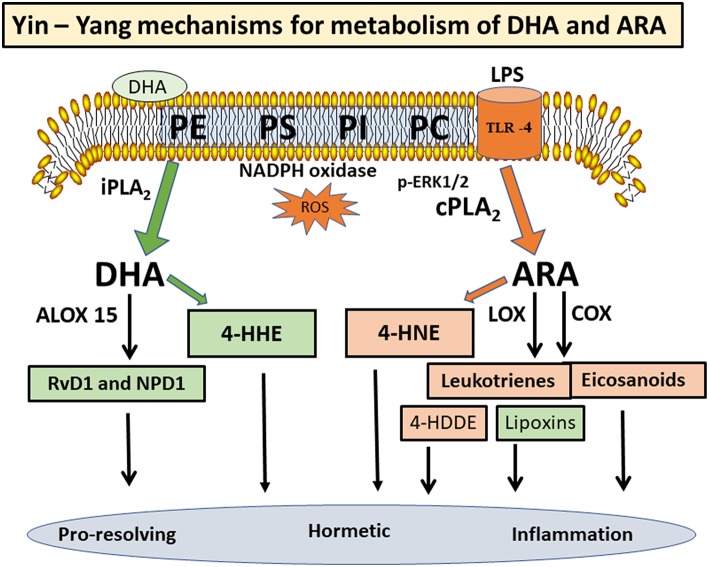
Yin-Yang mechanisms for metabolism of DHA, ARA, and their metabolites.

PUFAs are substrates for a number of oxygen enzymes including cyclooxygenases (COX), lipoxygenases (LOX), cytochrome P_450_ (CytP_450_), soluble epoxide hydrolase, and prostaglandin dehydrogenase, resulting in the production of a wild array of lipid mediators (Figure 2). Depending on the cell types and conditions, these lipid mediators have shown protective and detrimental effects. ARA is converted to prostaglandin H2 (PGH2) through COX1/2, and in turn, PGH2 is further converted to prostanoids (prostaglandins and thromboxanes) with both inflammatory and anti-inflammatory properties (18, 45). ARA also serves as substrate for LOX 5/12/15 to produce leukotrienes and lipoxins, and these lipid mediators work through specific receptors to mediate a variety of responses (46, 47). Interestingly, in contrary to the leukotrienes which exert cytotoxic and inflammatory effects in blood cells, lipoxin A2 and lipoxin B4 (synthesized through 5- and 15-lipoxygenases) are mediators with anti-inflammatory and immunomodulatory properties (see overview by Parkinson) (48). In a study by Livne-Bar et al, metabolomics screening for neuroprotective signals in astrocyte conditioned medium indicated the ability for lipoxin A4 and B4 to suppress injury-induced damage in retina (49). In another study on an intracerebral hemorrhage model in rat, lipoxin A4 was shown to offer anti-inflammatory effects and ameliorated the ischemic injury (50).

DHA is metabolized by a specific LOX (e.g., 15-lipoxygenase, Alox15) to form oxylipin intermediates leading to synthesis of resolvin D1 (RvD1), maresins, and neuroprotectin D1 (NPD1) (Figure 2). These structurally distinct molecules possess pro-resolving and pro-homeostatic properties (51–56). RT-PCR and Western blot analysis indicated high Alox 15 expression in the prefrontal cortex, olfactory bulb, and the hippocampal brain area (51). A study with human neuroblastoma cells demonstrated upregulation of Alox15 in association with histone deacetylases (HDAC), thus suggesting an epigenetic link (57). Aspirin-triggered RvD1 was shown to mitigate inflammation by enhancing the pro-resolution status as well as protecting the brain from synaptic dysfunction (58). Systemic prophylaxis treated with aspirin-triggered RvD1 was shown to improve surgery-induced cognitive decline and abolish synaptic dysfunction (59). Studies by Bazan's group indicated ability for NPD1to rescue inflammatory responses by inhibiting production of pro-inflammatory cytokines such as interleukin-1β (60). In a laser-induced mouse model, intraperitoneal injection of NPD1 could inhibit choroidal neovascularization, further confirming the mechanism for NPD1 to upregulate the resolution phase of the inflammatory response (61). Taken together, despite a Yin-Yang mechanism for metabolism of ARA and DHA, considerable cross-talks may occur among the lipid mediators in conferring detrimental and pro-resolving actions in different cell types and systems.

## Lipid Peroxidation of ARA and DHA

### 4-Hydroxy-alkenals

PUFAs, including DHA, ARA, linoleic acid, and linolenic acid, are susceptible to lipid peroxidation mediated by oxygen free radicals generated from a number of factors, including radiation, cellular response to xenobiotics, cytokines, bacterial and viral invasions, and mitochondrial oxidative stress (62, 63). A growing body of research has demonstrated the important role of lipid peroxidation in disease pathology, including diabetes, atherosclerosis, cardiovascular diseases, as well as neuroinflammation and neurodegenerative diseases (64–68). Oxygen free radicals can target the esterified PUFAs in membrane phospholipids as well as the free PUFAs in the cell cytoplasm. Peroxidation of these PUFAs is generally comprised of three main steps: initiation, propagation, and termination (69). These lipid peroxides are highly reactive, and upon interaction with iron, they can induce ferroptosis, which is a form of cell death different from that mediated by caspases (70, 71).

Depending on the position of the double bond, lipid peroxidation of n-3 and n-6 PUFAs can undergo fragmentation at different sites to give rise to a large number of carbonyl-containing lipid oxidation compounds including α, β-hydroxy aldehydes, acrolein, glyoxal, and malondialdehyde (72–74). The most prominent products are 4-hydroxyhexenal (4-HHE) from DHA and 4-hydroxynonenal (4-HNE) from ARA (75). In addition, ARA can react with 12-lipoxygenase to produce 12-hydroperoxy-eicosatetraenoate (12-HpETE), and in turn, undergoes peroxidation to form reactive 4-hydroxydodecadienal (4-HDDE) (69, 76, 77). These reactive aldehydes can readily undergo oxidation and reduction, and converted to acids or alcohols, which are more stable metabolites (78). Due to the high reactivity, these alkenal compounds are regarded as “second messengers of free radical reactions” (79), and biomarker of oxidative stress in inflammatory diseases [see review by Ito et al. (68)].

**Figure 3:**
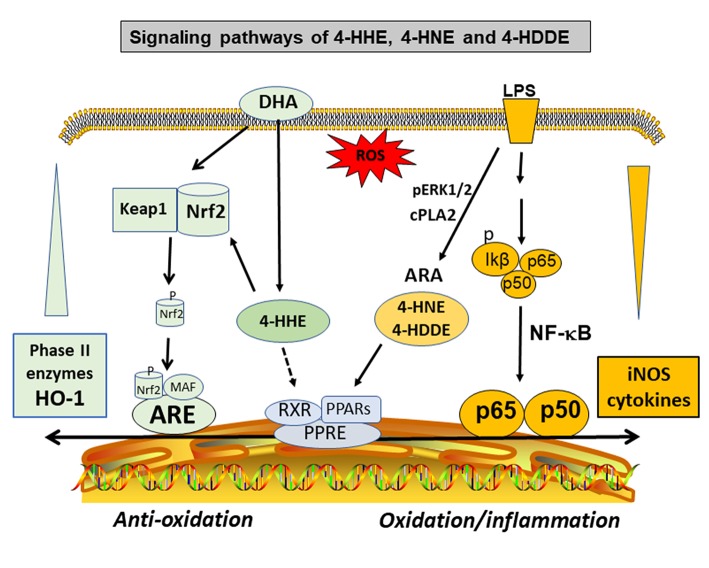
Hormetic and signaling effects of 4-hydoxy alkenals involving inhibition of NF-κB and cPLA_2_/ARA pathways, and activation of PPARs and Nrf2/HO-1 pathways.

An important property of the 4-hydox-alkenal products is their ability to form adducts with proteins, phospholipids, and nucleic acids through Michael addition and Schiff base reactions (73, 74, 77, 78, 80–83). The phospholipid adducts are formed largely with phosphatidylethanolamine (PE), especially the alkenylacyl PE subtype (77). These alkenal compounds are present widely in the body organs, in different cell types (84), and in body fluids including plasma and urine (76). Studies by Sasson's group provided evidence for binding of 4-HNE and 4-HDDE to peroxisome proliferator-activated receptors (PPARs). The activation of PPARs leads to a mechanism for their hormetic and regulatory effects against the pathology of metabolic and vascular diseases such as diabetes and atherosclerosis (69, 85–88) (Figure 3). Other studies demonstrated formation of protein adducts with 4-HNE in other metabolic and inflammatory diseases (78), such as rheumatoid arthritis (89), neuroborrelious (90), and in age-related disorders and low back pain (91, 92).

The involvement of 4-HNE and 4-HDDE in inflammatory diseases has led to studies searching for mechanisms regulating the release of ARA. Studies with microglial cells demonstrated the ability for lipopolysaccharides (LPS) to stimulate ARA release through phosphorylation of ERK1/2 and cPLA_2_ (15, 18), and in turn, stimulation of this pathway leads to an increased production of 4-HNE (84). Activation of Toll-like receptors by LPS also induces COX-2, which acts on ARA to produce prostanoids. A study by Uchida demonstrated the involvement of 4-HNE in the induction of COX-2 expression (through the p38MAPK pathway), implicating the role for 4-HNE in a feed-forward mechanism to enhance inflammation (93). The acyl groups in cardiolipin (CL) are comprised with high levels of PUFAs (especially linoleic acid), and is thus a good source for production of 4-HNE. Therefore, peroxidation of CL and production of 4-HNE could account for oxidative disturbances of mitochondrial function (6, 94).

The high levels of DHA in the brain and retina have alerted studies on the pathophysiological role of its peroxidation product, mainly 4-HHE [see review by Long et al. (95)]. However, despite similar properties for both 4-HNE and 4-HHE, subtle differences were observed. For example, in a study with primary neurons in culture, application *in vitro* of both 4-HHE and 4-HNE showed differences in toxicity with LD_50_'s of 23 and 18 μmol/L, respectively (96). In this study, cytotoxicity mediated by both alkenals could be suppressed by adding thiol scavengers such as glutathione (GSH) and N-acetyl cysteine, suggesting their ability to form adducts with these compounds. The study by Soulage et al. observed increase in circulating 4-HHE levels in type 2 diabetic human and in the diabetic Zucker rats (97). In this same study, exogenous 4-HHE was shown to reduce the glutathione concentration in L6 muscle cells (97). In a review by Long and Picklo, 4-HHE was 5- to 20-fold more potent in forming adducts with glutathione as compared with 4-HNE (95). Besides interaction with the GSH pathways, recent studies provided evidence for 4-HHE to exert hormetic effects by stimulating the stress response pathway involving nuclear factor-like 2 (Nrf2) and synthesis of anti-oxidant enzymes such as heme oxygenase-1 (HO-1) (95, 98–100) (Figure 3). In fact, our recent study with microglial cells also indicated ability for 4-HHE to induce higher levels of HO-1 as compared to 4-HNE (84).

Despite of perceived hormetic effects, the alkenal compounds may have deleterious and toxic effects, especially when tested at high concentrations and at *in vitro* settings. In a study by Awada et al. increase in 4-HHE in plasma was observed in mice consuming a diet with oxidized lipids, and the increase in 4-HHE was linked to oxidation and inflammation in the small intestine (101). In this study, the damaging effect on intestinal absorption was attributed to ability for 4-HHE to form protein adducts and alter glutathione metabolism (101). However, in a review by Mauerhofer et al., oxidized phospholipids could offer protective effects, including activation of PPARs, suppression of the toll-like receptors, and up-regulation of the Nrf2 anti-oxidant pathway (102). These diverse results clearly suggest the importance of future studies to further examine oxidized phospholipids and specific protein adducts formed by the reactive hydroxyl-alkenal compounds under different pathological conditions (103).

## Methods for Analysis of 4-HHE and 4-HNE

### Immunohistochemistry Assay of Protein Adducts

The reactivity of 4-HHE and 4-HNE to form adducts with biomolecules suggests the possibility to detect the adducts by antibody-based methods (73). An immunoblot assay was used to detect 4-HNE in human blood (104). In a study in which plasma proteins were separated by two dimension electrophoresis, increases in HNE-protein adducts were observed in autism subjects as compared to controls (105). Using a mouse monoclonal antibody that specifically recognizes the 4-HNE-histidine epitope, an ELISA method was developed for assaying 4-HNE in cell lysates (106), and increased levels of the 4-HNE-histidine adduct were observed in the middle frontal gyrus of AD patients as compared with controls (107). Using an anti-4-HNE adducted protein, increases in 4-HNE were observed in the hippocampus and entorhinal cortex in rat brain after binge ethanol administration (108). Due to the increasing interest to study the role of 4-HDDE, an oxidative by-product of ARA, a monoclonal antibody was recently developed against the 4-HDDE adduct (109). However, many of these antibody assays lack sensitivity and specificity, and commercial availability is limited (110). Therefore, future studies using advance techniques to detect specific protein adducts will enhance understanding the role of these peroxidation products in different disease conditions.

Efforts to develop immunohistochemical techniques to detect localization of 4-HNE in tissues and cells have also become important tools to understand the effects of oxidative damage in tissues and cells (111). In the study by Mamalis et al., increases in immunohistochemistry staining of 4-HNE together with other oxidant markers such as 8-hydroxydeoxyguanosine and advanced glycation end products, were detected in damaged human epidermis due to excessive exposure to UV light (112). In another study, age differences in 4-HNE immunoreactivity were observed in the mouse hippocampal CA1 area comparing groups at 40–42 to 50–59 weeks (113). In addition with an increase in oxidative stress with age, the increase in 4-HNE was marked by a decrease in mitochondrial dysfunction. Interestingly, administration of epigallocatechin-gallate, a polyphenol abundant in green tea extract, could attenuate 4-HNE immunoreactivity in the aged brain (114).

Relatively few studies have developed effective immune assay for 4-HHE. Using a mouse monoclonal IgG1 antibody, mAbHHE53, which is specific for protein-histidine bound HHE, increases in 4-HHE in autopsy samples of spinal cords were observed in amyotrophic lateral sclerosis patients (115). However, despite that the mouse monoclonal IgG1 antibody was developed to target 4-HHE, there is evidence that both 4-HNE and 4-HHE were detected (116). Considering that different PLA_2_s are responsible for the release of ARA and DHA and subsequently their peroxidation products, future studies for production of more specific immune products for the alkenals will help to advance understanding of their metabolism.

### LC-MS/MS Analysis for Simultaneous Measurement of 4-HHE and 4-HNE

Methods for accurate and precise analysis of free 4-HHE and 4-HNE present in biological samples include gas or liquid chromatography (GC or LC) and sometimes coupled with MS (73, 95). Depending on the chromophore, these peroxidation products could be quantified through LC with UV detection at 220 nm (117). However, due to lack of sensitivity and precision, GC-MS and LC-MS methods were developed as an alternative to the spectrophotometric detection. The classic multiple reaction monitoring assay performed on a triple quadrupole mass spectrometer has long been used for simultaneous quantification of 4-hydroxyalkenals, and this technique is capable of monitoring characteristic transitions for each targeted compound. Without derivatization, 4-hydroxyalkenals in human T cell leukemia extracts or mouse liver samples can be detected by selected reaction monitoring as [M+H] ^+^ (118, 119), but the sensitivity of this analysis is not sufficient to detect the levels of 4-hydroxyalkenals in small biological samples. Subsequently, different approaches were used to derivatize 4-hydroxyalkenals in order to increase detection sensitivity (120–124). Wang et al. enhanced sensitivity with a shotgun lipidomics-base method using the Michael adduct of 4-HNE with carnosine (122). Another agent is 1,3-cyclohexanedione (CHD), which takes advantage of the intrinsic reactivity of aldehydes, allowing for more specific and sensitive detection in different biological matrices (84, 100, 125–127). Because the alkenyl aldehydes are rather unstable, derivatization can help to stabilize these molecules. Recently, we applied the CHD-derivatization strategy to quantify the levels of 4-hydroxyalkenal species in different biological matrices over a wide range of concentrations (84, 128). Since phospholipids are common interfering species in reverse-phase LC, and can cause significant ionization suppression in the mass spectrometer, we employed a solid phase extraction (SPE) strategy to remove phospholipids while preserving the concentrations of the analytes (129). Our results for analysis of 4-HNE in biological samples, including plasma and brain tissue, fall into the range from 0.1 to 1.5 nmol/mg protein, depending on the samples and conditions (63, 120, 130, 131).

## Yin-Yang Mechanisms Regulating the Production of 4-HHE and 4-HNE

With an established LC-MS/MS protocol to simultaneously measure 4-HNE and 4-HHE, an experiment was carried out to examine factors regulating levels of the hydroxyl-alkenals in BV-2 microglial cells stimulated with LPS and/or supplemented with DHA (84). Results showed that treating cells with exogenous DHA led to a dose-dependent increase in 4-HHE but not 4-HNE, whereas stimulation of cells with LPS resulted in an increase in 4-HNE but not 4-HHE (84). Since LPS is known to stimulate cPLA_2_ and release of ARA in microglial cells (18), an association between 4-HNE and the cPLA_2_/ARA pathway was confirmed using inhibitors for cPLA_2_, which readily suppressed the LPS-induced increase in 4-HNE in these cells (84). Quercetin is a botanical polyphenol known to suppress LPS-induced cPLA_2_ and NF-κB related pro-inflammatory responses in microglial cells (132). Along this line, treatment of cells with quercetin also suppressed LPS-induced 4-HNE (133). Taken together, these studies demonstrated a link between stimulation of the cPLA_2_/ARA pathway and production of 4-HNE, and thus assay of 4-HNE can serve as a marker for the inflammatory pathway.

Our study with microglial cells indicated an increase in levels of 4-HHE upon treating microglial cells with DHA within a relatively short period of time of 6 hr (84). These results suggest that despite of uptake of DHA and incorporation into membrane phospholipids, some DHA may directly enter the cells and make available for lipid peroxidation to form 4-HHE. Studies by Ishikado and Nagayama with endothelial cells also attributed ability for DHA to exert anti-inflammatory effects through increased production of 4-HHE, and in turn, ability for 4-HHE to upregulate the antioxidant stress pathway with increased synthesis of HO-1 (125, 126). Consequently, the ability for DHA to suppress inflammatory responses through its oxidative product is a novel phenomenon worthy of future studies with animal models (Figure 3).

## n-3 PUFAs Supplementation and Lipid Peroxidation Products: Implications on Neurological Disorders

To-date, n-3 PUFAs in the form of fish oil are one of the most highly consumed dietary supplements by humans (134). Although fish oil contains substantial amounts of eicosapentaenoic acid (EPA) together with DHA, EPA is an intermediate for biosynthesis of DHA, and is present in relatively low levels in brain. In recent years, there is enormous interest to examine effects of dietary fish oil and DHA supplement on health and diseases [see review by Sun et al. (135)]. However, few studies have focused on the hormetic and deleterious effects of the peroxidative products. In a recent study, human subjects consuming Atlantic salmon showed increases in n-3 PUFAs in the phospholipids (e.g. PC) and triacylglycerols in plasma (136). In another study, healthy male subjects given different levels of DHA supplement (up to 1600 mg of DHA per day) showed a dose-dependent increase in levels of 4-HHE in the plasma (137). In agreement with the Yin-Yang mechanism for production of 4-HHE and 4-HNE, results of Calzada's study showed that DHA supplementation did not alter levels of 4-HNE. In this study, DHA supplementation not only did not alter redox homeostasis, but also prevented low-density lipoproteins from oxidation (137).

In a review by Trepanier et al. there is evidence for dietary n-3 PUFA to exert anti-inflammatory effects and neuroinflammatory outcomes in a number of animal models with neurological disorders (138). Studies with animal and cell models also pointed to the role of microglial cells in mediating the neuroinflammatory responses (139). Treating DHA to cells *in vitro* could mitigate LPS-induced pro-inflammatory responses through interaction with the toll-like receptor 4 (84, 140). Nevertheless, these effects *in vitro* are dependent on the treatment conditions, as high levels of DHA was shown to cause profound cell swelling and induce pyroptotic cell death (141). In a study with human microglial cells, both EPA and DHA were effective in decreasing the pro-inflammatory M1 markers and stimulating the anti-inflammatory M2 markers (142).

### Lipid Peroxidation in Cerebral Ischemia and Brain Injury

Although increase in oxidative stress has been implicated in many forms of brain injuries and ischemic stroke, relatively few studies have successfully assessed the peroxidation products under these conditions (143–145). In a study by Zhang et al., mice fed a fish oil diet showed resistance to insult due to focal cerebral ischemia. The protective effects of n-3 PUFA supplements were attributed to ability of 4-HHE to upregulate the antioxidant pathway involving Nrf2 and synthesis of HO-1 (146). Considering the important role for the Nrf2 pathway to promote transcription of a number of antioxidant genes for regulating the body defense systems, recognition of a metabolic link between DHA and/or 4-HHE with the Nrf2 pathway may provide therapeutic strategy against oxidative damage due to cerebral ischemia and other brain injuries (147, 148).

### Lipid Peroxidation in Neurological and Inflammatory Pain

There is emerging evidence for a connection between activated immune cells with oxidative stress, inflammation, and nociceptive responses (149–151). Low back pain is among the highest prevalence neurologic disorders in humans (152–154). Acrolein, a reactive aldehyde derived from a number of environmental factors, has been regarded as an important factor contributing to the hyperalgesia following traumatic injury. Besides acrolein, there is indication that other oxidant stressors such as 4-HNE and H_2_O_2_, may also augment the transient receptor potential channels (TRPA1 and TRPV1) in mediation of inflammatory responses and pain (155–157). More importantly, 4-HNE was shown to activate these channels through binding specific sites on the channel protein (158). In agreement with this phenomenon, inhibitors to acrolein and antibodies to oxidized phospholipids were able to mitigate the channel activity as well as diminish pain sensation (159). Future studies to further understand how the channel proteins are activated by these oxidant stressors will undoubted be an important path to advance therapy to mitigate neuropathic and inflammatory pain.

### Lipid Peroxidation Associated With Binge Alcohol Consumption

Indications that chronic alcohol consumption is linked to increases in oxidative stress in the brain have led to studies searching for the mechanism whereby ethanol may induce lipid peroxidation products (160). Studies by Collins' group used an animal model as well as an organotypic slice culture model to demonstrated a link between binge alcohol administration with increases in PLA_2_s together with PARP-1 and aquaporin-4 (AQP4) (108, 161, 162). Using a commercial antibody against 4-HNE protein adduct, binge ethanol administration was shown to induce increases in 4-HNE in specific brain regions, including the hippocampus and entorhinal cortex (108). In agreement with the increase in PLA_2_ activity, a time-dependent increase in levels of 4-HNE adduct was also observed in the brain slice culture experiment exposed to ethanol (161). This experiment further showed that supplementation of DHA (25 μM) to the slice culture could readily abrogate the oxidative markers due to the binge ethanol treatment (108). Consequently, future studies are needed to elucidate the mechanism for DHA to inhibit oxidative stress due to binge alcohol administration.

### Lipid Peroxidation Associated With Alzheimer's Disease (AD)

Oxidative stress is known to play an important role in AD pathogenesis; lipid peroxidation together with the increases in production of 4-HNE as well as 4-HHE have been reported in the progression of this disease (163–166). Oligomeric Aβ could target lipid peroxidation resulting in increase in 4-HNE which in turn, form adducts with cysteine, histidine, and lysine residues (167, 168). Accumulation of HNE-adducts may lead to irreversible changes and thus impairing metabolic functions in the development of AD pathology (169). In an AD transgenic mouse model, administration of oligomeric Aβ resulted in an increase of free PUFAs together with levels of 4-HNE in the brain, suggesting increase in lipid peroxidation activity (14). Since lipid peroxidation is a relatively early event of oxidative stress, increases in alkenal compounds were detected in the hippocampal gyrus in individuals in the preclinical phase (165, 170). In addition, increased levels of 4-HNE and acrolein were observed in the brains of mild cognitive impairment and in early AD patients (131). In the late AD stage, propagation and amplification of oxidative stress were marked by increases in malondialdehyde (MDA), acrolein, and 4-HNE (165).

In a group of AD patients showing age-related decline, DHA supplements appeared to show improvement in learning and memory in this group of patients (171). Dietary supplement of DHA also protected AD-model mice from amyloid and dendritic pathology (172–174). In a study to examine effects of DHA supplementation on different phospholipids in brain regions of AD mouse model, results indicated significant increases in PC and PE levels in cortex, hippocampus and cerebellum (175). However, further studies are needed to examine whether the changes in brain lipids due to dietary DHA supplementation are associated with altered lipid peroxidation activity in the brain.

### DHA and Lipid Peroxidation Associated With Autism Spectrum Disorder (ASD)

As reviewed by Romano et al. lipid peroxidation appears to play a role in several neuropsychiatric disorders, including bipolar diorder, depression, schizophrenia and Huntington's disease (176). Autism spectrum disorder (ASD) is a neurodevelopmental disorder that occurs in the early stage of life. The pathogenesis of ASD remains controversial; nevertheless, interactions between multiple genes and environmental risk factors are strong candidates (177). Early studies showed alterations in FA profiling in plasma of ASD patients with specific reduction of DHA (178). A study on proteomes in plasma of ASD and control subjects indicated significant increase in proteins involved in acute inflammatory response together with protein adducts to 4-HNE, thus suggesting a link between oxidative stress and lipid peroxidation in the pathophysiology of ASD (105). In particular, younger children with autism seem to be more vulnerable to oxidative stress and thus showing a greater increase in lipid peroxidation products. In a study on Egyptian autistic children, levels of malondialdehyde (MDA) were significantly higher in children with autism <6 years of age, whereas this difference diminished with increasing age of the children (179). In another study with older patients, significantly higher levels of 4-HNE protein adducts were found in erythrocyte membranes and plasma from autistic patients as compared with controls (180).

In a study with a gene/stress mouse model, maternal DHA supplementation was shown to mitigate autistic behaviors in offspring pups (181). In order to investigate mechanism(s) whereby DHA supplementation could suppress autistic behavior, Yang et al. examined FAs and peroxidation products in mouse pups from mothers given a control diet or a diet containing 1% DHA (128). Results showed increases in DHA and decreases in ARA levels in all regions of the pup brain. However, DHA-supplemented diet increased 4-HHE levels mainly in the cerebral cortex and hippocampal regions, whereas levels of 4-HNE were not changed (128). Considering the important role of neurons in mediating memory and cognitive functions in the cerebral cortex and hippocampus, the specific increase in 4-HHE in these brain regions upon supplementation with DHA suggests greater peroxidation activity associated with these brain regions. Further investigations are needed to examine the physiologic role of 4-HHE in brain development. This dietary regimen also altered DHA, ARA, and 4-HHE in mouse heart and plasma, and interestingly, analysis of these alkenals in plasma indicated not only an increase in 4-HHE but also a decrease in 4-HNE. A study by Nakagawa et al. showed a similar increases in DHA and decreases in ARA as well as elevated levels of 4-HHE but not 4-HNE in adult mice fed dietary fish oil in multiple peripheral organs of adult mice (100). Consequently, more studies are needed to examine whether increase in 4-HHE due to maternal DHA supplement can enhance the hormetic effects during brain development (88).

## Concluding Remarks and Future Perspective

The abundance of DHA and ARA in phospholipids in the CNS remains a subject of interest for understanding the physiologic role of their metabolites. The current review demonstrated a Yin-Yang mechanism for metabolism of DHA and ARA due to their release by different PLA_2_s, and these mechanisms led to diverse down-stream pathways for production of lipid mediators that are pro-inflammatory and pro-resolving.

Peroxidation of DHA and ARA by oxygen free radicals results in production of 4-HHE, 4-HNE, 4-HDDE, and other carbonyl products, which can be detected in body organs and body fluids. These reactive aldehydes are electrophiles, and can confer cytotoxic and protective effects depending on the conditions for production. There is increasing evidence for the hormetic and anti-inflammatory effects of these alkenals through interaction with PPARs, suppression of NF-κB inflammation, and upregulation of the stress pathway involving Nrf2 and synthesis of HO-1. However, more efforts are needed to identify binding of these alkenals to specific proteins in pathological conditions. Many neurological disorders are accompanied by oxidative stress, neuroinflammation and apoptotic cell death. Although it is not possible to elucidate individual mechanism(s) for the pathogenesis of these disorders, this review provided an emphasis on an increase in 4-HHE due to supplementation of DHA, and the increase in 4-HNE associated with the inflammatory pathway involving activation of cPLA_2_ and release of ARA. Due to the complexity of membrane lipids, advance techniques for lipidomics are needed to better understand mechanisms of oxidative stress and source of lipid peroxidation products in disease processes.

## Author Contributions

BY and GS initiated writing this review. KF, DB, ZG, JL, WF, and CG contributed substantially to the concept and editing of the review.

### Conflict of Interest Statement

The authors declare that the research was conducted in the absence of any commercial or financial relationships that could be construed as a potential conflict of interest.
